# Mesonephric adenocarcinoma of the vagina: Diagnosis and multimodal treatment of a rare tumor and analysis of worldwide experience

**DOI:** 10.1007/s00066-016-1004-x

**Published:** 2016-06-27

**Authors:** Iris Mueller, Gerhard Kametriser, Volker R. Jacobs, Gerhard Bogner, Alfons Staudach, Horst Koch, Pia Wolfrum-Ristau, Christiane Schausberger, Thorsten Fischer, Felix Sedlmayer

**Affiliations:** 1 Department of Obstetrics and Gynecology (OB/GYN), Paracelsus Medical University (PMU), Müllner Hauptstr. 48, 5020 Salzburg, Austria; 2 Department of Radiology and Radiation Oncology, Paracelsus Medical University (PMU), Müllner Hauptstrasse 48, 5020 Salzburg, Austria

**Keywords:** Quality of life, Diethylstilbestrol, Brachytherapy, Radiochemotherapy, Radiotherapy, Lebensqualität, Diethylstilbestrol, Brachytherapie, Radiochemotherapie, Strahlentherapie

## Abstract

**Background:**

Mesonephric adenocarcinoma of the vagina is an extremely rare tumor of the female genital tract, with only a few cases reported so far worldwide. Consequently, there is no established standard treatment and limited knowledge about the prognosis and biologic behavior of vaginal mesonephric adenocarcinoma.

**Methods:**

This report documents a new case of vaginal mesonephric adenocarcinoma diagnosed in a 54-year-old woman, and analyzes this in the context of all previously published cases.

**Results:**

MRI demonstrated that the 2.5 × 1.8 cm tumor of the vaginal wall was invading urethra and bladder. Following surgical excision, histologic analysis determined mesonephric adenocarcinoma of the vagina, stage pT2 R1. In order to avoid the mutilating extended surgery which would be required to reach R0 and considerable impairment of quality of life, adjuvant radiochemotherapy was administered with external radiation and brachytherapy, including 5 cycles of cisplatin (40 mg/m²) for radiosensitization. After 4 years of continuous oncologic follow-up, the patient is alive and clinically free of disease.

**Conclusion:**

In this case it was shown that adjuvant radiochemotherapy with radiation and brachytherapy was effective to manage the surgical R1 situation and maintain the patient’s life quality. More published cases reports are needed to gradually substantiate optimal treatment strategies.

## Zusammenfassung

**Hintergrund:**

Das mesonephrische Adenokarzinom der Vagina ist ein äußerst seltener Tumor des weiblichen Genitaltrakts. In der internationalen Literatur finden sich nur wenig gut dokumentierte Fälle. Das biologische Verhaltensmuster dieses Tumors und dessen Prognose sind weitgehend unbekannt.

**Methoden:**

In diesem Bericht wird ein neuer Fall einer 54-jährigen Frau mit einem mesonephrischen Adenokarzinom der Vagina vorgestellt und unter Berücksichtigung aller bisher publizierten Fälle analysiert.

**Ergebnisse:**

Bei dem 2,5 × 1,8 cm großen Tumor der Vaginalwand zeigte sich in der Magnetresonanztomographie eine Infiltration der Urethra und Harnblase. Es wurde eine Exzision des Tumors vorgenommen. Histologisch ergab sich ein mesonephrisches Adenokarzinom der Vagina im Stadium pT2 R1. Eine R0-Resektion wäre nur durch ein aggressives chirurgisches Vorgehen möglich gewesen. Um diese massive Einschränkung der Lebensqualität zu vermeiden, wurde eine adjuvante Radiochemotherapie mit externer Bestrahlung und Brachytherapie mit 5 Zyklen Cisplatin (40 mg/m²) zur Strahlensensibilisierung durchgeführt. Die Patientin ist im 4‑Jahres-Follow-up beschwerdefrei und ohne Rezidivgeschehen.

**Schlussfolgerung:**

In diesem Fall ist die adjuvante Radiochemotherapie mit Brachytherapie die bestmögliche Strategie, um auf die chirurgische R1-Situation einzugehen und dabei die Lebensqualität der Patientin zu erhalten. Weitere Fallberichte sind nötigt, um optimale Behandlungsstrategien zu etablieren.

## Background

Mesonephric adenocarcinoma (MA) of the vagina is a rare tumor of the female genital tract, with only six cases listed in MEDLINE as of March 2016 (Table 1). A few more reports on MA occurring in the cervix, uterus, and urinary bladder, including pediatric patients, have been published [1–3, 6, 7, 14, 16].

**Table 1:** Clinical characteristics of patients with mesonephric adenocarcinoma of the vagina reported in the literature. (Modified from Bifulco et al. [2])

	Index case	Bifulco et al. [2]	Hinchey et al. [16]	Bague et al. [3]	Ersahin et al. [1]	Roma AA [6]	Amal et al. [17]
Age (years)	54	58	29	54	55	58	50
Previous surgery	No	TAH + BSO	No	No	VH	SCH	n.k.
Symptoms	Vaginal bleeding	Pelvic pain, pruritus vulvae	Pelvic fullness	“Leiomyomas”	Asymptomatic	Vaginal bleeding	Vaginalbleeding
Size (cm)	2.5 × 1.8	14 × 7 × 6	6 × 6 × 4	4	0.9 × 0.6 × 0.5	5 × 2.5 × 2.5	n.k.
Surgical treatment	ResTu	ResTu + Pelvic + paraaortic LFN	ResTu + BSO	TAH + BSO + colpectomy	Radical upper vaginectomy BSO + pelvic LFN + sampling periaortic LFN	Pelvic exenteration + ileal conduit	Surgery(unspecified)
Adjuvant treatment	BT + Cis	No	Radiotherapy	No	BT + cisplatin	No	Yes (unspecified)
Follow-up	NED 4.5 years	NED 12 months	NED 4 months	AWD 8.7 years	NED 36 months	1 month	n.k.

*ResTu* resection of the tumor, *AWD* alive with disease, *BSO* bilateral salpingo-oophorectomy, *LFN* lymphadenectomy, *NED* no evidence of disease, *TAH* total abdominal hysterectomy, *BT* brachytherapy, *VH* vaginal hysterectomy, *SCH* supracervial hysterectomy, *n.k.* not known

The first link between adenocarcinoma of the vagina and intrauterine exposure to diethylstilbestrol (DES) was described in 1971 [11]. DES is an orally administered active synthetic estrogen which was routinely given to selected pregnant woman from about the 1940s to the 1960s, in the mistaken belief it would reduce the risk of pregnancy complications and losses. Follow-up studies demonstrated that exposure to DES in utero causes a spectrum of congenital anomalies in females, cervical and vaginal adenosis being those most commonly found [13]. MAs exhibit a variety of morphologic patterns and may therefore be confused with mixed Müllerian tumors and other neoplasms. Immunohistochemical findings may help to distinguish MAs from their Müllerian counterparts [2]. Many of the reported cases were previously categorized as mesonephric carcinoma and are now reclassified as clear-cell carcinoma of Müllerian type.

We present the case report of a 54-year-old woman with MA of the vaginal wall invading urethra and bladder and describe the course of treatment.

## Gynecologic case presentation

A 54-year-old Caucasian woman (1G 1P – 1 gravida, 0 para) was referred to the Obstetrics/Gynecology Department because of vaginal bleeding after cohabitation. During clinical examination, a tumor of size 2.5 × 1.8 cm located underneath the urethra was visible. In pelvic MRI, invasion of the urethra and the urinary bladder by the tumor was described, without any evidence of suspect lymph nodes. A cystoscopy showed no pathologic findings. Biopsy of the tumor revealed vaginal adenosis with microglandular hyperplasia. However, the presence of an adenocarcinoma could not be excluded. Local excision of the lesion was performed in general anesthesia (ITN). Histopathologic workup confirmed infiltration of the urethra and of parts of the muscularis of the urinary bladder. Microscopically, the tumor showed a tubulocystic and papillary pattern, with scanty pale to eosinophilic cytoplasm with low nuclear atypia. The immunohistochemical profile of the tumor cells was positive for pancytokeratin and CK7, but for calretinin and vimentin only focal positive. Furthermore estrogen and progesterone receptor were negative and CD 10 was slightly positive.

The final diagnosis was MA of the vagina, stage pT2 R1. Achievement of an R0 resection would only have been possible by extended, mutilating surgery resulting in a severely impaired quality of life. Hence, in the interdisciplinary tumor board the consecutive decision was made to treat in a conservative approach by adjuvant radiotherapy including sensitization with cisplatin in analogy to treatment strategies for gynecological adenocarcinoma [8].

## Radiotherapy

The planning target volume comprised the vulva, vagina, and the whole uterus, as well as bilateral inguinal, external, and internal iliac lymph nodes up to the pelvic inlet as the cranial field border. CT-based 3D-treatment planning was performed in supine position. External beam radiotherapy (EBRT) was delivered as intensity-modulated radiation therapy (IMRT; step-and-shoot, daily image guidance) with 15-MV photons on a linear accelerator (Elekta Synergy; Elekta, Stockholm, Sweden) [9], in single fractional doses of 1.8 Gy (5 fractions/week) up to a total dose of 48.6 Gy. EBRT was followed by fractionated high-dose rate (HDR) brachytherapy delivered by a vaginal applicator. The entire length of the vaginal mucosa including the whole introitus was treated with an additional overall surface dose of 22 Gy in two treatment courses in weekly distance, each of them comprising three fractions of 3.5 Gy (bid) or 4 Gy (once daily) on two consecutive days. Radiosensitization was performed with concomitant chemotherapy consisting of five cycles of cisplatin (40 mg/m²) once per week throughout the EBRT course, up to an overall dose of 329 mg at an individual body surface area of 1.67 m², according to common schedules for adjuvant treatment regimens in cervical carcinoma and following the suggestions of a previous case report [7]. The patient is alive and clinically free of disease at 4 years of follow-up.

## Discussion

Mesonephric adenocarcinoma of the vagina is a rare entity and any knowledge about is it therefore very limited. Prognosis, biologic behavior, and treatment strategies are controversially discussed and due to the rareness of the disease, there is no established standard therapy. Moreover, the histopathologic findings are complex and immunohistochemical studies show different results [5, 12]. Therefore, many cases previously categorized as MA have been reclassified as clear-cell carcinomas or malignant mixed Müllerian tumors, and vice versa [17].

The Müllerian and Wolffian ducts originate from mesodermal tissue. These ducts develop in both male and female embryos. In females the Müllerian ducts develop to form the Fallopian tubes, uterus, cervix, and upper two-thirds of the vagina; whereas in males they regress. Conversely, the Wolffian ducts form the male internal genitals but atrophy in females due to the absence of testosterone [10]. MAs may arise out of these remnants [15].

Compared to the malignant mixed Müllerian tumor, it seems that MAs located in the female genital tract have a better prognosis [2].

However, the optimal management strategy for MA remains uncertain. Treatment recommendations can evolve only gradually, based on episodic reports and, in principle, derived in analogy to allegedly comparable oncologic constellations. Radical surgery is suggested as a potentially curative option, particularly when malignant lesions appear well capsulated [3]. In this patient, RO resection would have only been possible by extended surgery with subsequent functional losses. Therefore, it was decided to treat by radiochemotherapy in a multimodal approach, in analogy to therapeutic approaches for frequent gynecological adenocarcinomas, e. g., vaginal cancer [4].

## Conclusion

Despite a limited current follow-up time of 4 years, this favorable disease course supports the hypothesis that these rare tumors are sensitive to adjuvant chemoradiation. Adjuvant treatment should be considered in cases with a higher probability of microscopic remnants, thus potentially also avoiding mutilation without compromising oncologic outcome.
